# Widespread domain-like perturbations of DNA methylation in whole blood of Down syndrome neonates

**DOI:** 10.1371/journal.pone.0194938

**Published:** 2018-03-30

**Authors:** Peter Henneman, Arjan Bouman, Adri Mul, Lia Knegt, Anne-Marie van der Kevie-Kersemaekers, Nitash Zwaveling-Soonawala, Hanne E. J. Meijers-Heijboer, A. S. Paul van Trotsenburg, Marcel M. Mannens

**Affiliations:** 1 Department of Clinical Genetics, Academic Medical Center, Amsterdam, The Netherlands; 2 Department of Pediatric Endocrinology, Emma Children’s Hospital, Academic Medical Center, Amsterdam, The Netherlands; 3 Department of Pediatrics and Translational Genetics, Emma Children’s Hospital, Academic Medical Center, Amsterdam, The Netherlands; University of Bonn, Institute of Experimental Hematology and Transfusion Medicine, GERMANY

## Abstract

**Introduction:**

Down syndrome (DS) is the most frequent genetic cause of intellectual disability. Despite the fact that more than 50 years have passed since the discovery of its genetic aberrations, the exact pathogenesis of the DS phenotype has remained largely unexplained. It was recently hypothesized that the DS pathogenesis involves complex (epi)genetic, molecular and cellular determinants. To date, many reports have addressed epigenetic aberrations associated with DS at different developmental stages/ages and tissue types, but to our best knowledge not in DS newborns. This study aimed to investigate genome-wide methylation patterns in DS newborns compared to non-trisomic newborns.

**Method:**

We analyzed blood samples obtained from ten newborns with DS and five age-matched non-trisomic newborns. Epigenetic profiles were obtained from extracted DNA using the Illumina Infinium 450K array. Since aberrant blood cell distribution is known to be present in DS, we applied two distinct models: with and without correction for estimated blood cell distribution.

**Results:**

Differentially methylated position (DMP) analysis of the uncorrected model detected 19525 significant hits (51,2% hypomethylated). In the corrected model, we found 121953 significant DMPs (49,8% hypomethylated). Independent of the used model we observed a chromosome 21 dosage effect. Moreover, we detected 46 and 145 differentially methylated regions in the uncorrected and corrected model respectively, both showing hypomethylation overrepresentation. Replication analyses of DMPs and DMRs found by Bacalini *et al*. (2015) showed a large overlap.

**Conclusion:**

In this study, we found methylation profile differences between DS newborns and controls reflecting a systematically affected epigenetic profile. The observed chromosome 21 dosage effect suggests the involvement of affected essential regulatory factors/regions or altered expression of chromatin modeling enzymes located on chromosome 21. Additional research is necessary to substantiate these hypotheses.

## Introduction

Trisomy 21, also known as Down syndrome (DS), is a common autosomal aneuploidy occurring in approximately 1 in 750 live births[1]. The DS phenotype includes a broad range of clinical features, like characteristic dysmorphisms, short stature, congenital heart and gastrointestinal disorders, a higher risk of having congenital or autoimmune thyroid disease, and intellectual disability[2]. The severity of the DS phenotype, however, is quite variable. Although more than 50 years have passed since the discovery of an extra chromosome 21 being the cause of DS, the precise aberrant molecular and cellular mechanisms underlying the DS phenotype and its variability have remained largely unknown[3]. It has been hypothesized that having 50% more chromosome 21 Down Syndrome Critical Region (DSCR) genes causes most of the phenotype (dosage effect hypothesis)[4, 5]. However, investigation of patients with different segmental trisomy 21 to identify and relate genes within the DSCR to specific DS features explained only part of the phenotype [6–8]. Just a few years ago a novel hypothesis was formulated, involving epigenetics. Since then, several studies have shown genome-wide aberrant DNA methylation profiles in DS, suggesting that DS phenotype may not be explained by over-expression of chromosome 21 genes only, but probably involves altered expression of genes on other chromosomes as well. Genome-wide DNA methylation has been studied in various DS tissues: blood, buccal epithelium, brain, placenta, skin, muscle, lung, and kidney[9–18], and results show that methylation differs between tissues and changes over time. Since DNA methylation is not static but dynamic, abnormal methylation patterns in DS adults may be influenced by a lifetime of exposure to environmental factors [13, 19, 20]. In addition, recent studies in fetal DS tissues show that the prenatal epigenetic status is different from both the adult and neonatal statuses[9, 21]. To minimize the influence of (postnatal) environmental factors on DNA methylation, we studied genome-wide methylation in ten DS newborns shortly after birth compared to five non-DS newborns of the same age.

## Materials and methods

### Study samples

We obtained blood DNA samples from ten newborns, in whom for patient care reasons, QF-PCR and karyotyping had been performed. QF-PCR was performed with the QSTR-plus, v2-kit of Elucigene (Manchester, United Kingdom) for chromosomes 13,18, 21, X and Y. The manufacturer-protocol was used for analysis of all patients. All ten patients showed a complete trisomy 21 with QF-PCR (Table A in S1 File). DS by QF-PCR was confirmed by karyotyping (mean cell count: five) to exclude that the trisomy was familial. All ten patients showed free trisomy 21 (Fig. A in S1 File). Samples were made available anonymously, but we were informed about the age at blood sampling (mean age: day 1; range: day 0–3) and gender (seven boys and three girls). Simultaneously, we obtained five age-matched anonymous control samples with a normal karyotyping result (one boy and four girls). Further clinical data of the controls were not available. DNA was isolated from whole blood (heparin). Bisulfite-treatment (ZYMO®) was applied to genomic DNA. For each sample, DNA methylation profiles were obtained by the Illumina infinium 450K DNA methylation array. Randomization was employed in all experimental batches. Technicians performing laboratory experiments were blind to clinical data (DS or normal karyotype). All analyses were performed using R (version: 3.2.2, running under Ubuntu version 15.10). The study was approved by the Academic Medical Center Research Ethics Board (CCMO: NL45496.018.13).

### Data quality and normalization

Primary quality control (QC) on the raw 450K dataset was performed using the R shiny package MethylAid (version 1.4.0)[22]. MethylAid is a user- friendly visual and interactive web application enabling detection of bad quality samples using sample-dependent and sample-independent control probes present on the 450K DNA methylation array. The present study solely applied default MethylAid thresholds, i.e., methylated and unmethylated intensities (MU) of 10,5, overall quality control (OP) of 11,75, bisulfite control (BS) of 12,75, hybridization control (HC) of 13,25, and a detection P-value (DP) of 0,95. Next, we normalized our 450K dataset using quantile normalization, implemented in the Minfi package (version 1.16.0).

### Epigenome-wide association

We applied the Minfi package for secondary quality control and genome-wide screening of DNA methylation variation associated with DS[23]. Prior to the association analyses, probes located on the sex chromosomes, and SNP associated probes (CpG, Probe and SBE minor allele frequencies >0,05) were removed from the dataset. Density plots before and after quantile normalization were evaluated. In order to estimate the contribution of gender-related and other possible confounding batch effects, a principal component analysis was performed. DS is associated with immunological dysfunction and deficiencies and the imbalance of leukocyte cell types distribution in DS has been described extensively[24, 25]. Estimation of leukocyte cell distribution was performed within the Minfi package according to the method of Houseman *et al.[26]*. This method was originally designed to overcome analysis bias due to an imbalance of blood cell type by coincidentally occurring immunological reactions in individuals within a study cohort. Houseman algorithm is based on 450K signatures and generally yields information on relative counts of CD8+ and CD4+ T cells, natural killer cells, B cells, monocytes, and granulocytes. Because aberrant blood cell distribution in DS might be related to our factor of interest, namely DNA methylation, we addressed this duality by applying two models: (i) DNA methylation = group + gender and (ii) DNA methylation = group + gender + CD8 + CD4 + NK + Bcell + MonoC + Gran. Both models were applied in the differentially methylated probes (DMPs) analysis using the *lmfit* function (method = robust) and in differentially methylated regions (DMRs) analysis using the “bumphunter” function. DMP and DMR outcomes were used in all further evaluations. General methylation differences were evaluated by counting the probes with a δ < 0, representing hypomethylation and counting the probes with a δ > 0, representing hypermethylation, both concerning the total number of probes present in the dataset. In addition, we evaluated hypo- and hypermethylation among the genome-wide significant probes (q<0,05). Detection of DMRs (“bumphunter”) was done by using the following settings: difference cutoff, 0,2; bootstrapping, 500 (iterations); smoothing, loessbycluster L>2. For evaluation of DMRs about domains associated with dysregulation of gene expression, the LaminB1track (Tig3; Human diploid embryonic lung fibroblast cells) was downloaded and merged with our DMR outcomes reported in S1 Table. Detailed information of the track is available at: https://genome.ucsc.edu/cgi-bin/hgTrackUi?hgt_tSearch=Search&g=lamin B1Super and the report of Guelen *et al*.[27].

### Candidate gene association

In addition to a hypothesis-free approach, i.e., a genome-wide evaluation of DMPs and DMRs, we followed a hypothesis-driven approach. For the latter, we used the results of a recent study on DS associations by Bacalini *et al*.[28]. Their study design was similar to ours concerning DNA source (whole blood) and models adjusting for cell distribution or not, but differed concerning age and relationship between cases and controls. The main difference between our study and the study of Bacalini *et al*. was the age of the DS individuals and matching controls, i.e., Bacalini *et al*. used adult DS samples, and siblings or mothers as controls. Association results of candidate probes were extracted from the whole epigenome dataset, and compared with the data of Bacalini *et al*. Only probes that met the most stringent criteria according to Bacalini *et al*. (n = 68) were evaluated. For evaluation of the direction of effect, i.e., concordance between studies about hypo- and hypermethylation, P-values were used. Multiple test correction was applied, according to the Benjamin-Hochberg method; probes with a q < 0,05 were assumed significant. Bacalini *et al*. also reported on enrichment of differentially methylated genes in four main categories: i) hematopoiesis (*RUNX1*, *DLL1*, *EBF4*, *PRMD16*), ii) morphogenesis and development (*HOXA2*, *HOXA4*, *HOXA5*, *HOXA6*, *HHIP*, *NCAM1*), iii) neuronal development (*NAV1*, *EBF4*, *PRDM8*, *NCAM1*, *GABBR1*), and iv) regulation of chromatin structure (*PRMD8*, *KDM2B*, *TET1*). These sets of genes were used in our study for replication analysis as well, using similar criteria about the direction of effect and significance.

## Results

### Data quality and normalization

**Fig 1:**
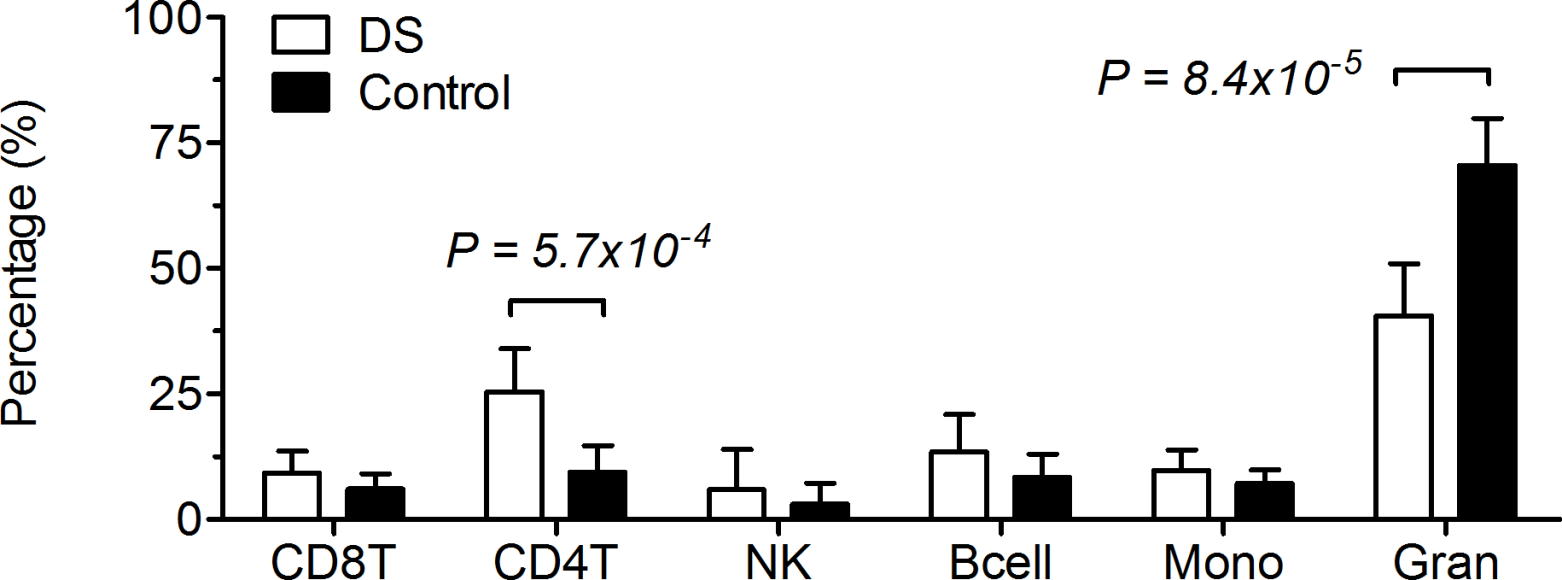
Estimated blood cell distribution. Relative estimated distribution of CD8+ and CD4+ T cells, natural killer cells, B cells, monocytes, and granulocytes among down syndrome (DS) and controls.

Initial quality control using MethylAid analysis showed no poor quality samples concerning hybridization, bisulfite, or overall quality controls. Subsequently, we removed probes annotated to the X -and Y chromosomes (11648), probes involving a SNP at the CpG or extension sites (51710), probes prone to cross-hybridization (1125) and non-specific probes (22570). In total, we removed 87053 probes yielding a total of 398459 probes entering further analyses. As expected, subsequent raw data quantile normalization showed good density profiles (S1A Fig). To investigate the presence of confounding technical or biological batch effects, we performed Principal Component Analysis (PCA). S1B Fig shows the first component vs. the second, with DS cases and healthy controls. We observed clearly separated clusters for the cases and the controls, indicating systemic DNA methylation differences reflected by the first two Principal Components. Since gender distribution of cases and controls was not ideally matched, subsequent PCA was done with males and females annotated by color (S1C Fig). The PCA did not show a clear confounding effect of gender. Nevertheless, in further analyses, gender was applied as a covariate in the models. Next, we estimated the distribution of the most abundant cell types in human blood in DS cases and controls. Fig 1 illustrates the relative contribution of T-cells (CD4+ and CD8+), natural Killer Cell, B-cells, monocytes, and granulocytes. Significant differences in relative concentrations of CD4+ T-cells (P = 5,7x10^-4^) and granulocytes (P = 8,4x10^-5^) were observed; CD4+ T-cells were more abundant in cases than in controls, while the relative granulocyte count in cases was decreased. These observations indicated that blood cell type distribution was strongly related to our factor of interest (DS). In general, blood cell distribution correction is a good method to remove this kind of unwanted biological variation. However, since blood cell type distribution is known to be affected in DS [24, 25, 29], we assumed that neither a model with blood cell correction nor a model without blood cell correction would be ideal. Therefore, we decided to apply and report both models.

### Epigenome-wide association DMPs

**Fig 2:**
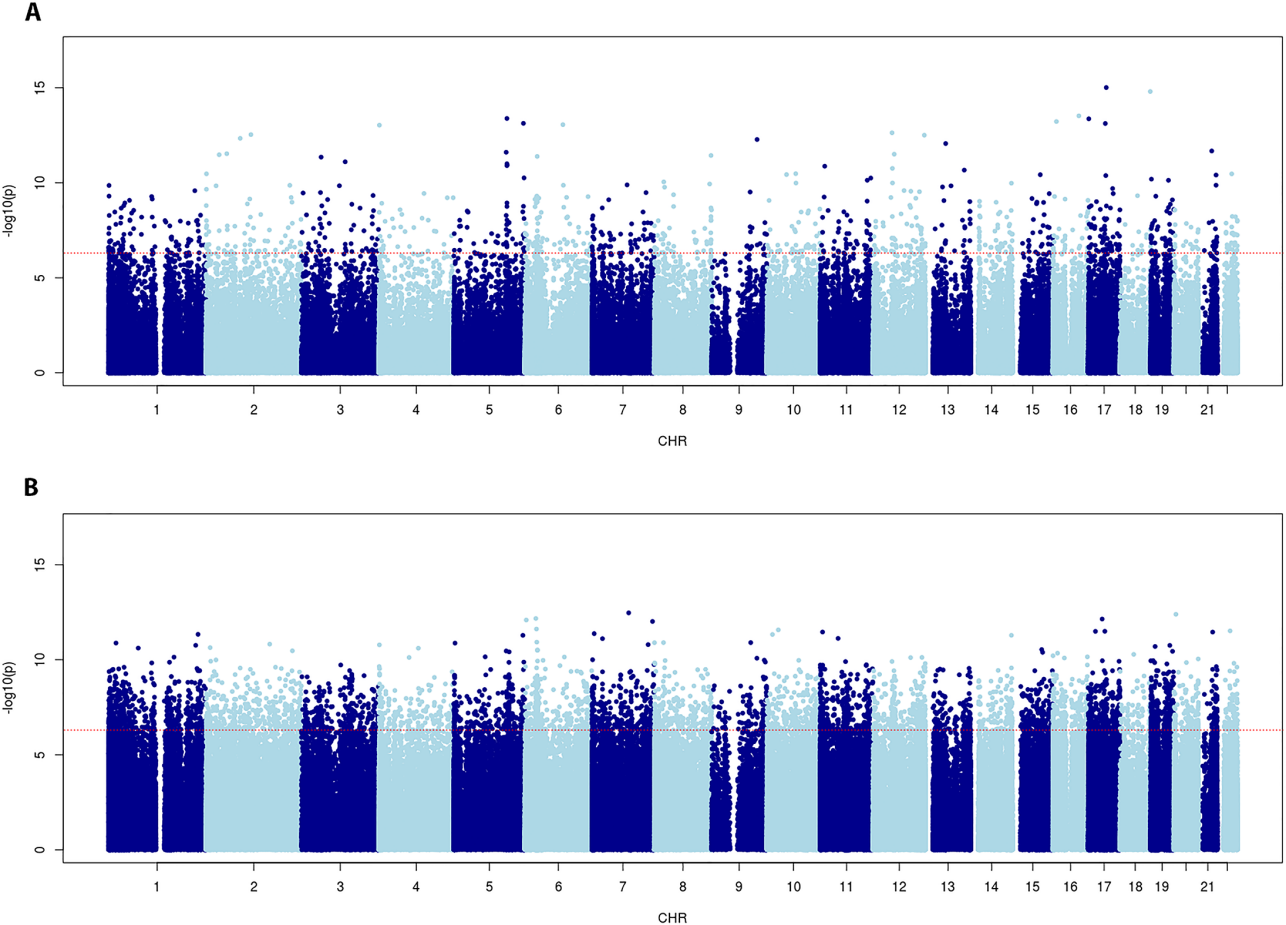
Manhattan plots. (A) Manhattan plot DMP association analysis (probes associated with SNP, MAF>0,05, and probes located on X and Y chromosomes were excluded from analysis), corrected for gender but not for blood cell distribution. X-axis represents ranked chromosomes, Y-axis represents–log10 (P-value). *Redline* indicates significance level (Bonferroni; P < 5 10^−7^). (B) Manhattan plot DMP association analysis (probes associated with SNP, MAF>0,05, and probes located on X and Y chromosomes were excluded from analysis), corrected for gender and blood cell distribution. X-axis represents ranked chromosomes, Y-axis represents–log10 (P-value). *Red line* indicates significance level (Bonferroni; P < 5 10^−7^).

**Fig 3:**
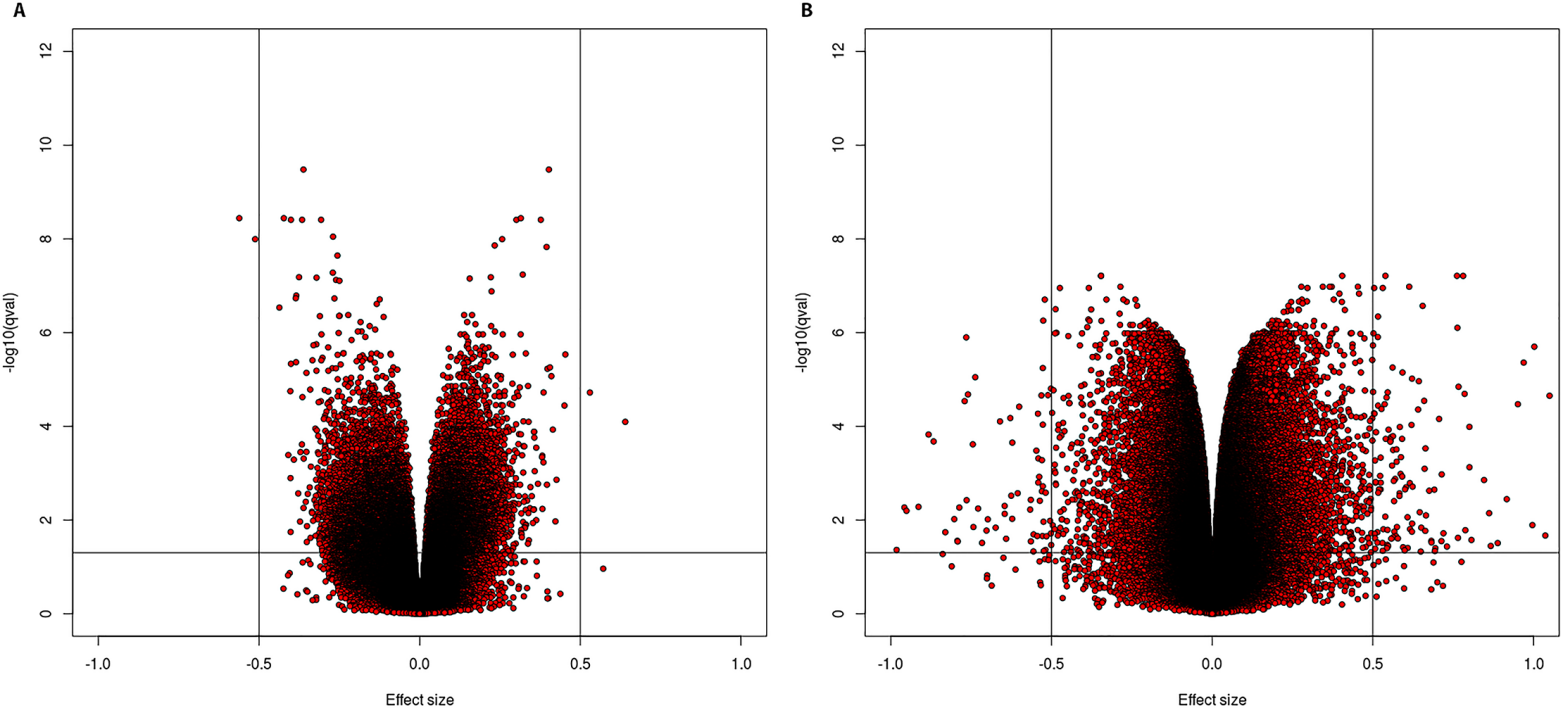
Volcano plots. (A) Volcano plot DMP association analysis (X and Y chromosomes were excluded from analysis), corrected for gender but not for blood cell distribution. X-axis represents (effect size) adjusted mean delta difference (δ based on adjusted coefficients), Y-axis represents–log10(q-value). *Horizontal line* indicates significance level (P < 5 10^−7^*)*, *vertical lines* indicate δ_abs_ > 0,5. (B) Volcano plot DMP association analysis (X and Y chromosomes were excluded from analysis), corrected for gender and blood cell distribution. X-axis represents (effect size) adjusted mean delta difference (δ based on adjusted coefficients), Y-axis represents–log10(q-value). *Horizontal line* indicates significance level (P < 5 10^−7^*)*, *vertical lines* indicate δ_abs_ > 0,1.

**Fig 4:**
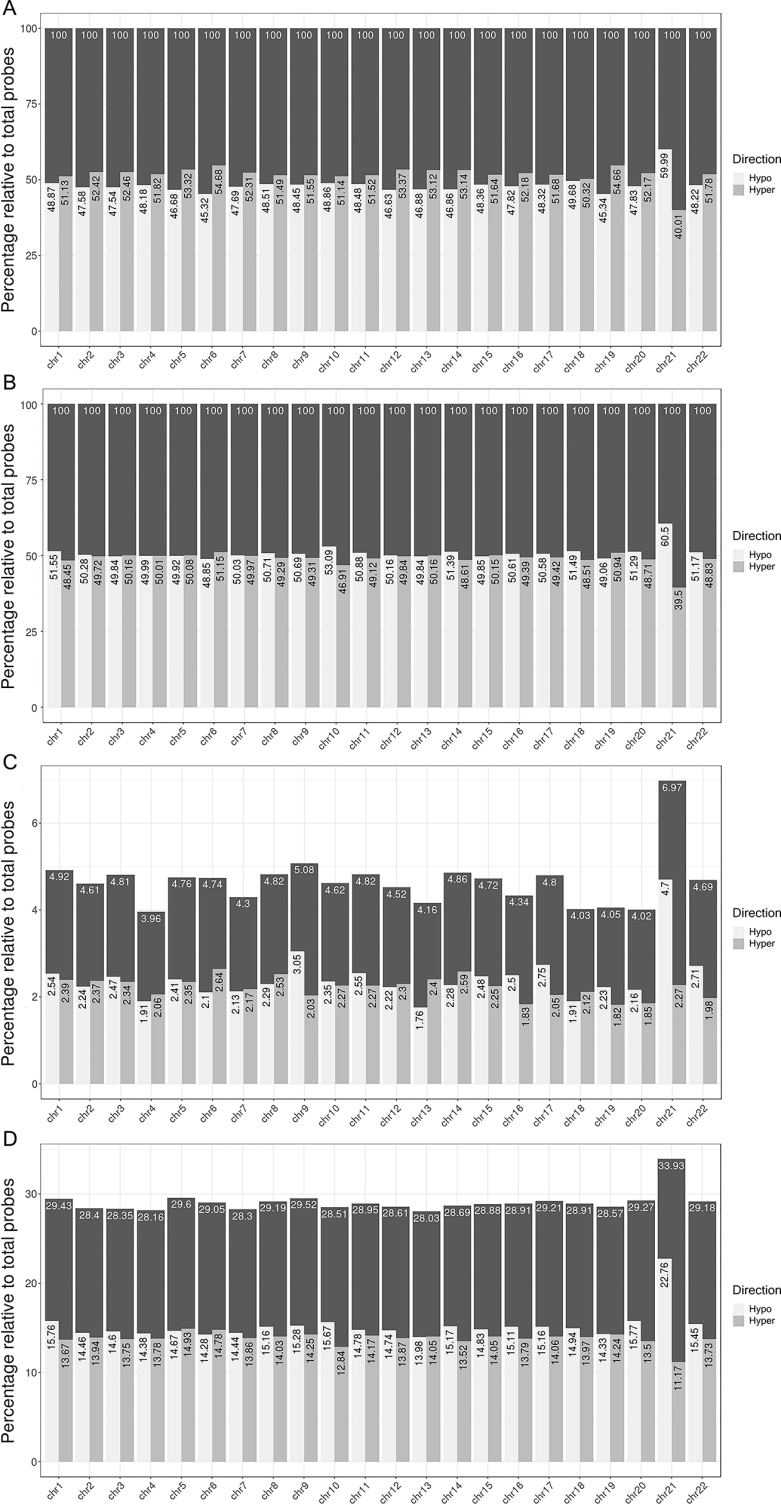
Distribution of hypo -and hypermethylation over the autosomes in DS vs. controls. (A) Relative to the total number of probes, percentages of hypo- and hypermethylated probes. Hypomethylated δ < 0, hypermethylated δ > 0. DMP association analysis corrected for gender but not for blood cell distribution. (B) Relative to the total number of probes, percentage of hypo- and hypermethylated probes. Hypomethylated δ < 0, hypermethylated δ > 0. DMP association analysis corrected for gender and blood cell distribution. (C) Relative to the total number of genome-wide significant (q<0,05) probes, percentage of hypo- and hypermethylated probes. Hypomethylated δ < 0, hypermethylated δ > 0. DMP association analysis corrected for gender but not for blood cell distribution. (D) Relative to the total number of genome-wide significant (q<0,05) probes, percentage of hypo- and hypermethylated probes. Hypomethylated δ < 0, hypermethylated δ > 0. DMP association analysis corrected for gender and blood cell distribution.

**Fig 5:**
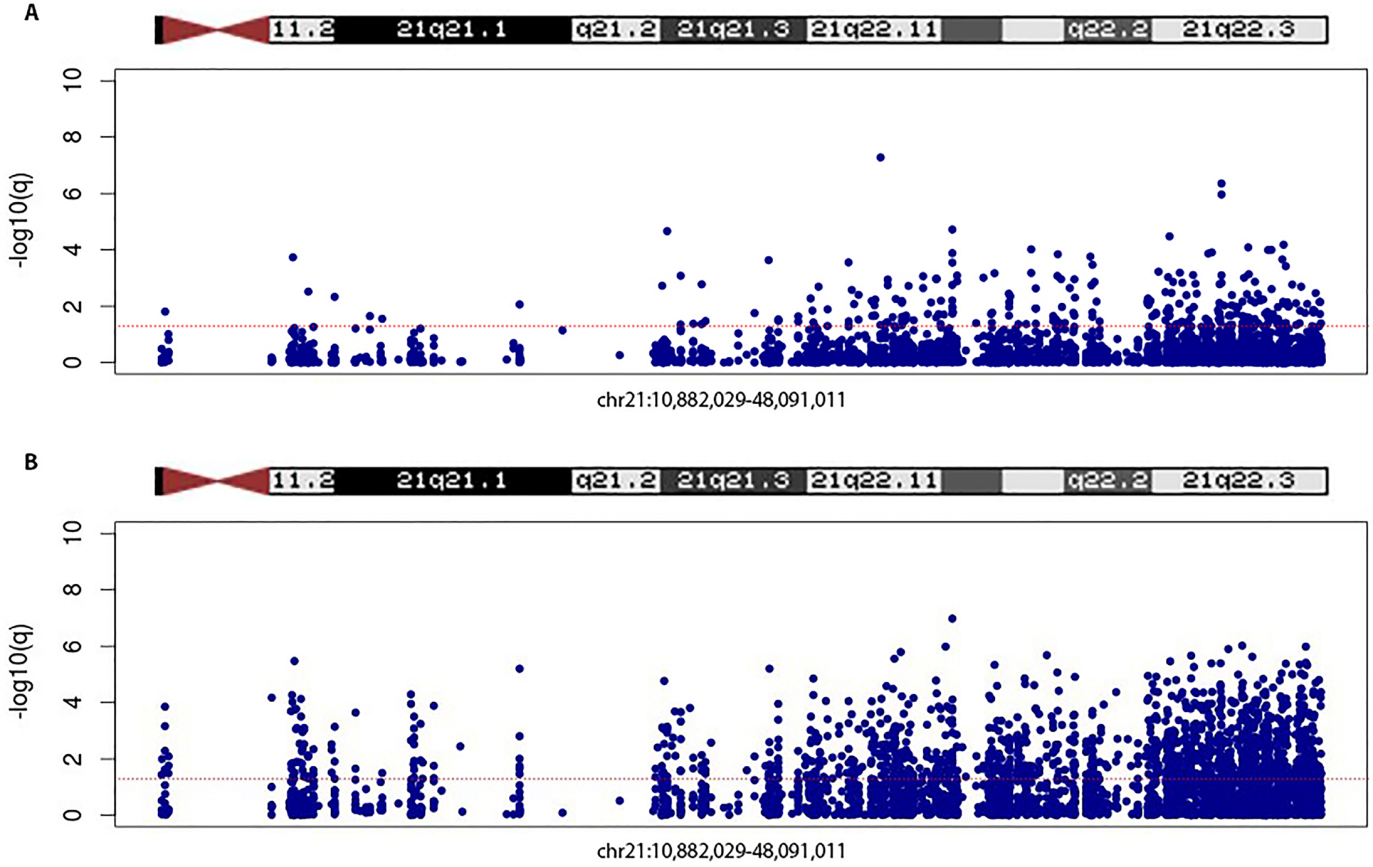
Chromosome 21 Manhattan plots. (A) Manhattan plot chromosome 21 only, DMP association analysis, corrected for gender. X-axis represents ranked position chromosome 21, Y-axis represents q-value DMPs. *Red line* indicates significance level (q < 0,05). (B) Manhattan plot chromosome 21 only, DMP association analysis, corrected for gender and blood cell distribution. X-axis represents ranked position chromosome 21, Y-axis represents q-value DMPs. *Red line* indicates significance level (q < 0,05).

Differentially methylated probe analysis was performed on the normalized dataset using *lmfit*, and applying two models: (i) corrected for gender, and (ii) corrected for gender plus blood cell distribution (T-cells (CD4+ and CD8+), natural Killer Cell, B-cells, monocytes, and granulocytes). In the model corrected for gender only, we found 19525 significant (q< 0,05) DMPs, of which 48,9% showed hypomethylation. In the model corrected for gender plus blood cells, we found 121953 significant DMPs, of which 51,7% showed hypomethylation (Table 1 and S1 Table). Manhattan plots, with chromosome position represented on the X-axis and -log(p-value) on the Y-axis for both models, are represented in Fig 2A and 2B. The red line in these plots indicates P< 10^−7^. Further evaluation about q-value and effect size in both models was performed using volcano plots (Fig 3A and 3B). In order to evaluate the specific distribution among all 22 chromosomes, we calculated the relative percentages of hypo- and hypermethylation per chromosome of all probes and the genome-wide significant probes, respectively. Fig 4A and 4B show the relative percentages of hypo- and hypermethylation about the total number of probes per specific chromosome for both models. We observed a clear enrichment of significant hypomethylated probes located on chromosome 21 in both the blood cell uncorrected model (59,9%), and blood cell corrected model (60,5%). In addition, we observed slightly higher percentages of hypermethylation throughout the other chromosomes in the blood cell uncorrected model, but not in the blood cell corrected model. In addition, we evaluated the relative percentage of hypo- and hypermethylation about the genome-wide significant (q<0,05) probes per specific chromosome only, for both models. Both models showed an enrichment of hypomethylation on chromosome 21: 4,74% vs. 1,77%-3,09%, and 22,76% vs. 14,29%-15,80% for the uncorrected and corrected model, respectively (Fig 4C and 4D). Interestingly, our data suggest an enrichment of significant hypomethylated DMPs in virtually all autosomal chromosomes in the blood cell corrected model, but we did not observe such enrichment in the uncorrected model (Fig 4C and 4D). Our data (Fig 4A–4D) does not only indicate hypomethylation specific for chromosome 21 but also enrichment of significant DMPs located on chromosome 21. Therefore, we addressed the question whether these significant DMPs were located in any specific region of this chromosome. Fig 5A and 5B illustrate a chromosome 21 specific Manhattan plots for both the blood cell uncorrected and corrected model, respectively. The Y-axis in these plots represents the -log10 (q-value), while the X-axis represents the ranked position on chromosome 21. Note that the p-arm of the chromosome is poorly covered by the 450K DNA methylation array. We did not observe a specific region on chromosome 21 enriched for genome-wide significant DMPs.

**Table 1:** Number of differentially methylated positions and regions between DS and controls.

	Gender	Gender + blood cells
DMP (q < 0,05)	19525	121953
Hypomethylated (N / %)	9550 / 48,9%	63092/ 51,7%
Hypermethylated (N / %)	9975 / 51,1%	58861 / 48,3%
DMR (L ≥ 3)	46	145
Hypomethylated (N / %)	29 / 63,0%	92 / 63,5%
Hypermethylated (N / %)	17 / 36,0%	53 / 36,5%
DMR (L ≥ 5)	5 / 10,9%	28 / 19,3%

### Epigenome-wide association DMRs

Next, we addressed the detection of differentially methylated regions (DMRs) in DS cases vs. controls using the “bumphunter” function. Table 1 describes the number of observed DMRs including more than 3 probes (L>3) in the blood cell uncorrected model (46), and in the blood cell corrected model (145). Both models show a clear relative overrepresentation of hypomethylated regions; 63,0% and 63,5% for the uncorrected and corrected model, respectively. For the uncorrected and corrected model, we found no evidence for enrichment of DMRs on chromosome 21. Detailed information on DMRs is described in S1 Table(tabs DMR BCuncorrected and BCcorrected model, respectively). Next, we evaluated the presence of domains associated with dysregulation of gene expression located at our detected DMRs, in particular on chromosome 21 since all the chromosome 21 DMRs mapped to the distal one-third of 21q. In the uncorrected model, we observed that 15 of the 46 (33%) detected DMRs were associated with a lamina-associated domain (LAD) and in the corrected model we observed that 44 DMRs of the 145 (30%) detected DMRs were associated with a LAD. We did not observe enrichment of DMRs at LAD annotated to chromosome 2,1 albeit that in the corrected model the DMRs annotated to the *RUNX1* and *C21orf56* genes both reflected a relatively strong LaminB1 log ratio value of -1,2 and -0,5 respectively. For the *C21orf56* gene two distinct DMRs were detected, i.e., one DMR located nearby the 1st exon and a second DMR located at the intronic gene body which is associated with a LAD.

### Candidate gene association

**Fig 6:**
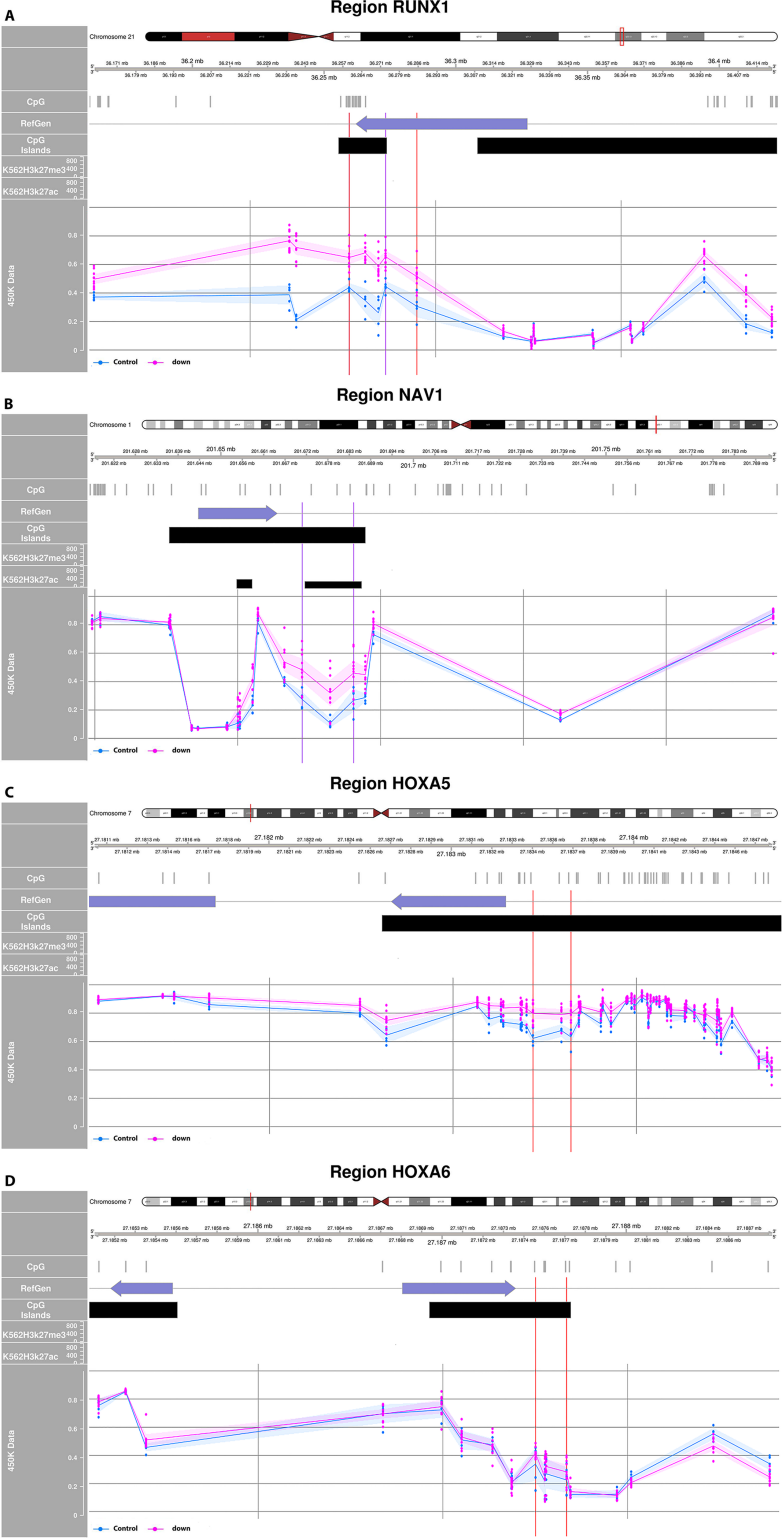
Regional plots of replicated DMRs. CpGs within DMRs are annotated between colored vertical lines. (A) *RUNX1*, overlapping (4 of 5 probes) DMRs detected in the uncorrected and corrected model are highlighted separately, (B) *NAV1*, DMR detected in the uncorrected model, (C) *HOXA5* and (D) *HOXA6*, for both genes, DMRs detected in the corrected model. X-axis represents the chromosomal position and Y-axis represents methylation index (Beta value). **Tracks:** *CpG*: localization of CpG sites (UCSC, HG19), *RefGen*: Reference gene, blocks represents exonic regions, arrow indicates direction of transcription (UCSC, HG19), *CpG island*: blocks represent the localization of CpG islands (> 300bp, GC content > 50%, ratio observed/expected CpG > 0,6). *K562 H3k27me3*: histone 3 lysine 27 3 methylation mark in K562 (chronic myelogenous leukemia human cell line, gain of the mark is associated with decreased activity of gene). *K562H3k27ac*: histone 3 lysine 27 acetylation mark in K562 (chronic myelogenous leukemia human cell line, an increased level of this mark is associated with increased gene activity, i.e., active enhancer mark).

In order to obtain DS candidate loci we performed a literature search using the terms [Down Syndrome AND Epigenetics] OR [Down Syndrome AND DNA methylation] which yielded 31 studies. Evaluation of these studies revealed that most were based on different tissue types and/or different detection platforms which would complicate replication analysis due to the large heterogeneity among the studies. After careful evaluation, we excluded most studies for our replication study with the exception of the recent report of Bacalini *et al*. (2015). The study of Bacalini *et al*. matched our survey very well about study design, analysis platform (Illumina 450K) and tissue type (DNA obtained from peripheral blood)[21]. In order to replicate previously reported findings observed in DS individuals we extracted the most stringently associated probe sets (regions, N = 68), covering a total of 203 individual probes reported by Bacalini *et al*. Moreover, strict thresholds were used in our evaluation of the replication, i.e., we used genome-wide adjusted q-values; q<0,05 was assumed significant. S2 Table illustrates the overlap between the results of Bacalini *et al*. and our survey, i.e., DMPs and DMRs in the uncorrected and corrected model, respectively. In total, 203 probes were evaluated of which 14 probes were omitted in our dataset due to QC filtering. Of the remaining 189 probes, 103 probes (54,5%) obtained by the uncorrected model and 115 probes (60,8%) obtained by the corrected model were also significant in our genome-wide survey. In addition, we observed that 180 (95,2%) of the remaining 189 probes showed concordant DNA hypo- or hypermethylation in DS individuals (see S2 Table, last column). In three probe sets we observed different methylation patterns in our survey compared to the survey of Bacalini *et al*. These three probe sets involved *FBXL16* (2 probes, max δ ≈ -0,02), *CPLX1* (2 probes, max δ ≈ -0,03), and *GABBR1* (5 probes, max δ ≈ -0,03). Bacalini *et al*. also reported DMRs located in sets of candidate genes that were selected because of involvement in essential biological functions, namely: i) hematopoiesis—*RUNX1*, *DLL1*, *EBF4* and *PRMD16*, ii) morphogenesis—*HOXA2*, *HOXA4*, *HOXA5*, *HOXA6*, *HHIP* and *NCAM1*, iii) neuronal development—*NAV1*, *EBF4*, *PRDM8*, *NCAM1* and *GABBR1*, and iv) regulation of chromatin structure—*PRMD8*, *KDM2B* and *TET*. Of these reported gene sets we observed four DMRs in our dataset (Fig 6A–6D, S2 Table). In the uncorrected model, we observed DMRs in the *RUNX1* and the *NAV1* genes, while in the corrected model we observed DMRs in the *RUNX1*, *HOXA5*, and *HOXA6* genes. Our data did not replicate Bacalini’s previously reported DS-associated DMRs located in the *HHIP*, *KDM3B*, *HOXA2*, *GABBR1* and *PRDMD8* genes (data not shown).

## Discussion

In this study, we found methylation profile differences between newborns with trisomy 21 and newborns with a normal karyotyping result, in DNA isolated from whole blood. Although previous studies have reported the same profile differences, to our best knowledge such a study has not been performed in newborns. Furthermore, many studies have focused on tissues like chorionic villi, placenta, brain, buccal cells and other tissues. Only a limited number of studies were performed in whole blood with adjustment for bias caused by differential cell type distribution in DS. Although the statistical power of the present study was expected to be low due to the small sample size and the heterogeneous phenotypic character of DS, we were able to detect thousands of DMPs and hundreds of DMRs associated with DS, and we observed a clear enrichment of these hits at chromosome 21. Furthermore, we replicated some findings by Bacalini *et al*. (2015). With the exception of the age and relationship of the cases and controls, their study design was quite similar to ours. Our study results confirm previous findings concerning the aberrant DNA methylation present in DS individuals about the methylation dosage effect of the extra chromosome 21. Since DNA methylation studies in DS have been performed in different tissues obtained prenatally and at several postnatal developmental stages, it is likely that, for many loci, aberrant DNA methylation is consistent. The consistent abberant DNA methylation indicates a systemic defect in gene expression regulation which is present from an early developmental stage into adult life. Possible explanations for such a systemically affected regulation could be an imbalance of expression of certain essential transcription factors, or a persistent aberrant chromosomal territory organization within the nucleus, which results in disturbed *in cis* and/or *in trans* interactions between chromosomal domains. Another explanation could be altered expression of chromatin modeling enzymes, such as DNA methyltransferases, resulting in a systemically affected epigenome in DS patients[15]. We suggest that DS may be designated as a ‘chromatin remodeling disorder’ with DNA methylation as a molecular fingerprint of this process. However, it is evident that additional studies are required to elucidate further such a mechanistic basis underlying the DS phenotype.

### 450K data-quality control and study design

Primary quality control (QC) of the 450K dataset showed bad data quality for none of the DS patients or controls. All submitted samples were therefore included in our statistical workflow for detection of differentially methylated positions and regions. Several genetic, epigenetic or phenotypic characterization studies were previously reported, but, to our best knowledge, epigenetic studies in DS newborns have not been described. A sampling of DS newborns and especially of healthy controls, is always a challenge about sample size. Although the sample size of this study was limited, this may not severely affect our statistical power, since the genetic and epigenetic heterogeneity in DS syndrome can be expected to be small compared to complex traits, such as diabetes mellitus or obesity. Gender differences in epigenetic studies, however, cannot be underestimated, and a limitation of our study is the imbalanced distribution of male and female subjects over the case and control groups. Moreover, sample sizes were too small for stratified analysis. Nevertheless, the PCA results did not show clear gender effects, nor did we observe association of any previously detected gender-specific locus, (data not shown)[30]. Furthermore, we used gender as a covariate in both statistical models, and excluded the X and Y chromosomes from the analysis. However, we could not stratify our analyses for gender, and thus we can only claim solely general effects.

### Statistical models

Tissue-specific epigenetic profiles are a well-known bias in epigenetic studies. DS shows a broad phenotype involving a variety of affected tissues, e.g. brain, heart, and blood. Thus, studying DS whole blood does not necessarily represent a surrogate tissue, but rather represents an important type of DS affected tissue. An aberrant blood cell type distribution is well described in DS patients[24, 25, 29]. Since every blood cell type can be assumed to be more or less specific in their epigenetic profile, this will result in a clear epigenetic difference between DS and controls but does not imply epigenetic differences between DS and controls *per se*. Studying both epigenetics and gene expression aberrations using the whole blood of DS patients, even when applying a blood cell distribution adjustment, remains therefore challenging. At the same time, blood cell distribution differences between individual subjects may confound the outcome of epigenetic surveys as well. Houseman *et al*. (2012) constructed a method to overcome individual blood cell distribution differences, making feasible adjustment for blood cell distribution. However, when blood cell distribution is strongly associated with the phenotype, as in DS, such adjustment might also affect the outcome of the analysis. For those reasons, we have used two models in our study: one without blood cell distribution adjustment and the other with blood cell distribution adjustment.

### DMP association analysis

For both the blood cell uncorrected and the blood cell corrected model, our survey for DMPs yielded a huge number of genome-wide significant hits: 19525 hits and 121953 hits, respectively. The fact that we detected this large number of hits among all chromosomes replicates findings of others and implicates a systemic differentially methylated state in DS compared to controls. The total number of significant hits differed between both models. The p-values of the Manhattan plot of the blood cell corrected model are somewhat dampened in comparison to the p-values in the plot of the uncorrected model, i.e., less extreme p-values are observed in the corrected model. The total number of hits, however, is much larger in the corrected model. The question whether the corrected model or the uncorrected model reflects a DS DNA methylation profile the best is difficult to answer. The corrected model might be prone to over-correction, while the results of the uncorrected model might relate to the difference in blood cell distribution rather than to a general DS DNA methylation profile. Overlap of both models’ top hits and, more importantly, replication of previously reported loci is therefore necessary for deciding which model is the best choice.

Several previous studies detected genes involved in chromatin structure as well. In particular, Jin *et al*. suggested that the genes *TET1* and *TET2*, encoding proteins involving the establishment of certain chromatin structures via DNA methylation or de-methylation, might play a role in DS. As a matter of fact, several DMPs located in the *TET1* and *TET2 genes* were genome-wide significantly associated with DS in both the uncorrected and the corrected model in our study. Since our study and studies of others indicated large genome-wide aberrations at DNA methylation level or gene expression level, *TET1* and *TET2*, or other epigenome editing enzymes, might indeed be good candidates for unraveling the underlying mechanisms of DS. The fact that some of these genes were replicated independently of sampled tissue or age enforces this suggestion of a systemic and early role of these genes. The exact mechanism by which the DS chromosomal aneuploidy trans-affects such DNA or protein modification genes is not yet known. The recent report of Mendioroz *et al*. (2015) described a study on such trans-effects of chromosome aneuploidies on DNA methylation in human DS and mouse models[18]. This study showed a large overlap of differentially methylated and hydroxyl-methylated positions between adult and fetal DS brain, confirming an early onset of these changes. Moreover, Mendioroz *et al*. showed that many of these altered DNA modifications involved transcription factor binding sites, which may lead to altered expression of these genes. The mechanism underlying this chromosome 21 aneuploidy trans-effect is still under debate.

### Global aberrant methylation

Considering all effect sizes, i.e., not applying any significance filter, we have not observed a general direction of the effect sizes, suggesting that there is no particular global enrichment of hypomethylation or hypermethylation present in DS vs. controls. About possible differences between the models we used, the volcano plots of both models clearly indicate that the corrected model yields far more genome-wide significant hits and yields generally larger delta differences, expressed as the model’s adjusted coefficient. The latter indicates that blood cell adjustment affects the significance and the value of the coefficients, which implies that blood cell distribution is, in fact, an important factor that should be addressed in epigenetic or gene expression studies of DS whole blood vs. healthy controls. Next, we addressed whether we could detect differences in hypo- and hypermethylation between DS and controls. Both models showed a deviation of the distribution of hypo- and hypermethylation (no filter applied on a threshold of delta differences) from what is expected by chance, i.e., approximately 50% of probes are hypo- and hypermethylated. Our evaluation of all probes and of the significant probes in both models showed consistent hypomethylation of probes located on chromosome 21. This finding is not in line with findings of some other studies. For example, Jones *et al*. (2013) did report a clear enrichment of hypermethylated significant hits (2190 hypermethylated vs. 1100 hypomethylated)[12]. One important difference between our survey and the survey Jones *et al*. is the type of tissue that was sampled. While our study was based on whole blood, Jones *et al*. reported on buccal epithelial cells. Another study on epigenetic differences between DS and controls was reported by Kerkel *et al*. (2010)[13]. Although this report did not involve a genome-wide survey, but rather an epigenetic survey on eight candidate genes, Kerkel *et al*. reported no clear statement about the global direction regarding all their studied candidates, which virtually reflects our observation. It is worth noting that the study of Kerkel *et a*l was based on leukocytes, which resembles our peripheral blood sample. The report of Jin *et al*. (2013) reported a genome-wide epigenetic survey based on reduced bisulfite sequencing of DS chorion villi and adult DS peripheral blood DNA[9]. This report showed large numbers of genome-wide significant hits, but, in contrast to the reports of Jones *et al*. and Kerkel *et al*., it also showed the presence of general hypermethylation in DS samples compared to healthy controls. In short, several reports show that a large number of loci are affected in DS patients, but about the direction of these effects, i.e., hypo- or hypermethylation, these reports do not point towards the same conclusion.

### DMR association analysis

Both our genome-wide approach and our candidate gene approach already indicated substantial epigenetic changes in DS vs. controls, showing strong clustered regions of DMPs. For the uncorrected model, we found 46 DMRs although none of these DMRs were genome-wide significant. For the corrected model we found 145 DMRs, six of which showed genome-wide significance (P-value < 3,45 x10^-04^). These six DMRs were located in or nearby the following genes: 1) *MIR886;* that encodes for a microRNA and has to our knowledge not been reported to be involved in any particular disease. 2) *EIF4E;* has been associated with autism and hypopharynx cancer, immunological disorders and DS. 3) *DUSP22;* has been associated with alkaline negative anaplastic large cell lymphoma and Duane syndrome, the latter involves an eye movement disorder. 4) *PM20D1;* which is linked to peptidase activity, but is, to our knowledge, not consistently associated with any particular disease. 5) The protein encoded by *NINJ2* promotes neurite outgrowth and has been associated with Acrocallosal Syndrome. This syndrome involves symptoms like aplasia/hypoplasia of the corpus callosum, postaxial hand polydactyly, and preaxial hand polydactyly. 6) *HDAC4*, encodes for histone deacetylase 4, which plays a critical role in transcriptional regulation, cell cycle progression, and developmental events. Histone acetylation or deacetylation alters the chromatin structure and affects transcription factor accessibility to DNA.

### Chromosomal dosage effects

In the present study, we used two statistical models and evaluation methods. The frequencies of hypo- and hypermethylation of all probes about the total number of analyzed probes per chromosome showed a trend towards general hypermethylation in DS vs. controls. However, this effect was not present in the corrected model, nor it was observed when evaluating only the genome-wide significant probes. We can conclude from these results that it is likely that no general hypo- or hypermethylation effect is present in DS and that blood cell type adjustment might be essential to overcome a certain bias in the hypo-/hypermethylation evaluation. Interestingly, from our evaluation of chromosome 21, we can conclude that there is a clear dosage effect on its DNA methylation status in the form of hypomethylation and the number of significant hits, independent of the used model. Our results on chromosome 21 hypomethylation are not in line with the findings of Jin *et al*. and Jones *et al*., which both performed genome-wide surveys. It is worth noting that the report of Jin *et al*. does claim overexpression of genes located on chromosome 21. Since hypomethylation is generally associated with increased expression, the observation of Jin *et al*. indirectly confirms our findings of consistent hypomethylation of chromosome 21 in DS. Our findings do, confirm the recently published results on general and dosage effects in DS patients, reported by Bacalini *et al*. It should be noted that the design and targeted tissue of this study was similar to our study. Further evaluation of the hits located on chromosome 21 addressed the question whether we could detect large specific regions that show aberrant DNA methylation in DS. Chromosome 21 specific Manhattan plots did not indicate that large specific regions show aberrant methylation. Therefore, we do not have a clear indication that specific regions located on chromosome 21 are associated with DS, although all detected chromosome 21 DMRs mapped to the distal one-third of 21q. However, it should be noted that the resolution of the array on specific arms of this chromosome is relatively low, making it hard to justify the latter claim.

### Differential methylation and genomic domains

In 2008, Letourneau *et al*. reported on genome-wide expression dysregulation domains (GEDDs) in DS patients vs. controls. In this study, lamina-associated domains (LADs) were associated with gene expression. Our DMR analyses might suggest the involvement of such domain-like regions as well. Although in the present study expression data was not available, *in silico* analysis of lamina-associated domains was feasible since such data is publicly available[27]. In this context, we focused on the LaminB1 mark that is associated with lamina-associated domains which generally associates with H3K4me3 marks, CTCF binding sites, promoters or CpG islands. Disturbance of the LAD is generally associated with altered gene expression, as was reported by Letourneau *et al*.[31]. Our analysis showed a substantial number of LADs present at DMRs detected in the uncorrected and corrected model, i.e., 15 of the 46 DMRs in the uncorrected model and 44 of the 145 DMRs in the corrected model. Although we cannot claim a true enrichment of LADs at our detected DMRs, the total number of LADs in the human genome is thought to be limited to approximately 1600 sites. This limited number of LADs favors the hypothesis that DS is characterized by genome- wide domain-like perturbations of DNA methylation but that these domains are not particularly enriched at chromosome 21 (*RUNX1* and *C21orf56*). Nevertheless, for the differentially methylated locus *C21orf56*, we detected a second DMR (trend genome-wide significant) annotated to the intergenic gene body near the first exon. Although this locus was not annotated to a LAD, it is associated with other regulatory marks (e.g. H3K27ac and DNase), which is in line with what is generally seen at LADs. Although our detected DMRs and their additional annotation to LADs confirm findings of others that reported on domain-like perturbations, these results have to be taken with caution since they are still based on *in silico* analysis of non-trisomy 21 tissues or cell lines other than whole blood. In that context, LADs can be placed under the umbrella of the so-called “epigenome” for which is known that it is cell and tissue-specific and strongly affected in cell culturing as well. Whether the observed relation between the publicly available cultured lung fibroblasts derived LADs data and our DMRs identified in whole blood represents a true association needs to be confirmed in additional studies, analyzing DNA methylation, expression and LADs within the same sample and in a combined analysis approach.

*DMP and DMR candidate evaluation*: In order to minimize a possible study design bias affecting our replication analyses, we only used the recent report of Bacalini *et al*. (2015)[9]. This study was also based on peripheral blood and, in addition, Bacalini *et al*. implemented a similar approach, with two different models, addressing the issue of blood cell distribution bias. Moreover, our choice for using only the study by Bacalini *et al*. seemed appropriate since their outcomes were discussed about other previously published DS association data, e.g. the studies of Kerkel *et al*. (2010) and Jones *et al*. (2013)[12, 13]. Here we report a large overlap between our DMP association results and the results of Bacalini *et al*., based on 203 individual probe loci, replicating > 50% of significant findings. Moreover, for >95% of the candidate probes that were evaluated, we observed concordant hypo- or hypermethylation between the two studies. Since our study was performed in DS newborns, these observations strongly suggest that for many loci affected in DS, the aberrant epigenetic status is consistent throughout (early) development and adulthood. Bacalini *et al*. also reported on several DMRs located in genes that potentially play an important role in mechanistic biological processes that are assumed to be involved in the manifestation of DS. We detected the presence of a DMR associated with DS located at the genes *RUNX1*, *HOXA5*, *HOXA6* and *NAV*,*1* which is partly in line with the findings of Bacalini *et al*. Nevertheless, we do confirm the involvement of the previously suggested biological processes in neuronal development, morphogenesis, and in particular hematopoiesis. About both the detected and replicated methylation differences on chromosome 21, it should be noted that Alves da Silva *et al*. recently showed that DMRs annotated to chromosome 21, i.e. the *RUNX1* but also the *WRB* genes, can be linked to their parental chromosomal origin[32]. In the present study, we were unable to study the parental origin of the extra chromosome 21 in our DS patients. This limitation implies that DNA methylation differences we detected for e.g. *RUNX1* might concern an imprinted locus which in turn may imply that these particular detected DMRs are rather a consequence of the parental origin than a true epigenetic aspect within DS.

In conclusion, in our study, we found methylation profile differences between DS newborns and non-trisomic newborns in DNA isolated from whole blood, reflecting a systemically affected epigenetic profile. Since such aberrant methylation profile has been observed in different tissues as well as at different ages, this indicates a persistently different epigenetic regulation in DS patients. The present study contributes to the hypothesis that chromosomal aneuploidies affect DNA methylation systemically and throughout the whole genome. The observation of a clear chromosome 21 dosage effect suggests the involvement of affected essential transcription factors encoded on chromosome 21, disturbed chromosomal territories and domains that systemically affect the epigenetic profile in DS, and/or altered expression of chromatin modeling enzymes, such as DNA methyltransferases. Additional research is necessary to substantiate these hypotheses. Raw and processed data of this study are available at GEO (www.ncbi.nlm.nih.gov/geo) under accession id: GSE107211.

## Supporting information

S1 File DS sample inclusion and script differential methylation analyses. Description of DS sample inclusion using QF PCR and karyotyping. Description of the “R” script used for differential methylation analyses.
**Table A in S1 File. QF PCR in DS patients.** QF PCR peak areas of D21S11, D21S1435, D21S1437, D21S1442 and D21S1446 markers in all DS patients.
Fig. A in S1 File. Karyotyping in DS patients.
(DOCX)

S1 Table Differential methylated positions. (XLSX)

S2 Table Replicated differential methylated positions and regions. (XLSX)

S1 Fig Data quality control. (a) Density plot raw and normalized (Quantile) data; (b) Plot principal component analysis (PCA), component 1 (20.2%) vs. component 2 (18.8%); component 3 (7.8%) and component 4 (6.3) are not shown. Colour annotation represents cases (red: Down syndrome) and controls (black healthy controls). X and Y chromosomes were excluded from analysis.; (c) Plot principal component analysis (PCA), component 1 (20.2%) vs. component 2 (18.8%); component 3 (7.8%) and component 4 (6.3) are not shown. Colour annotation represents genders (red: male, black: female); D and N represent Down syndrome and healthy controls, respectively. X and Y chromosomes were excluded from analysis.
(DOC)

## Abbreviations

DS: Down Syndrome
DMP: Differential Methylated Position
DMR: Differential Methylated Region
EWAS: Epigenome-wide Association Study
