# Supplementary material for: Widespread domain-like perturbations of DNA methylation in whole blood of Down syndrome neonates
Source: PLoS One. 2018 Mar 30;13(3):e0194938. doi: 10.1371/journal.pone.0194938 (PMC5877863; doi:10.1371/journal.pone.0194938)
Supplement: S1 File — Description of DS sample inclusion using QF PCR and karyotyping. Description of the “R” script used for differential methylation analyses. Table A in S1 File. QF PCR in DS patients. QF PCR peak areas of D21S11, D21S1435, D21S1437, D21S1442 and D21S1446 markers in all DS patients. Fig. A in S1 File. Karyotyping in DS patients. (DOCX) [file pone.0194938.s001.docx]

Widespread domain-like perturbations of DNA-methylation in whole blood of Down syndrome neonates

Peter Henneman^1^, Arjan Bouman^1^, Adri Mul^1^, Lia Knegt^1^, Anne-Marie van der Kevie-Kersemaekers^1^, Nitash Zwaveling-Soonawala^2^, Hanne E.J. Meijers-Heijboer^1^, A.S. Paul van Trotsenburg^3^, Marcel M. Mannens^1^

^1^Department of Clinical Genetics, Academic Medical Center, Amsterdam, The Netherlands, ^2^Department op Pediatric Endocrinology, Emma Children’s Hospital, Academic Medical Center, Amsterdam, The Netherlands. ^3^Department of Pediatrics and Translational Genetics, Emma Children’s Hospital, Academic Medical Center, Amsterdam, The Netherlands.

**Supplementary File 1.** DS sample inclusion and script differential methylation analyses

**DS patient inclusion**

QF-PCR was performed with the QSTR-plus, v2-kit of Elucigene (Manchester, United Kingdom) for chromosomes 13,18, 21, X and Y. The manufacturer’s protocol was used for analysis of all patients. All ten patients showed a complete trisomy 21 with QF-PCR (Table A). DS by QF-PCR was confirmed by karyotyping (mean cell count: five) to exclude that the trisomy was familial (Fig. A, below). Karyotyping was done by Q banding using quinacrine (QFQ) and G banding using giemsa and trypsin (GTG). All ten patients showed free trisomy 21.

**Table A:** QF PCR peak areas of D21S11, D21S1435, D21S1437, D21S1442 and D21S1446 markers in all DS patients.

|  | **D21S11** | **D21S1409** | **D21S1435** | **D21S1437** | **D21S1442** | **D21S1446** |
| --- | --- | --- | --- | --- | --- | --- |
| **DS_1** | 22225  0  0 ** | 11371  20275  0 * | 14369  7416  0 * | 30929  15010  0 * | 33205  16049  0 * | 9322  9240  9720 |
| **DS_2** | 15796  26511  0 * | 36906  0  0 ** | 13384  27168  0 * | 12921  25049  0 * | 22721  11037  0 * | 25681  24220  24753 |
| **DS_3** | 6852  5867  5636 | 12344  6370  0 * | 5638  4919  6067 | 5953  6868  6306 | 8366  7040  6218 | 12869  12930  4044 |
| **DS_4** | 10851  4863  0 * | 7637  12690  0 * | 9553  4169  0 * | 8147  16052  0 * | 14201  11699  12151 | 14585  6485  0 * |
| **DS_5** | 19107  17250  14881 | 44880  18140  0 * | 15478  28800  0 * | 15445  27066  0 * | 28906  28683  25245 | 53398  25038  0 |
| **DS_6** | 8157  8568  7949 | 35222  0  0 ** | 7967  12995  0 * | 12607  10324  9276 | 35405  17041  0 * | 31146  0  0 ** |
| **DS_7** | 4114  4117  3786 | 6394  5653  6025 | 4026  7150  0 | 24722  0  0 | 13284  12608  12383 | 11278  5070  0 |
| **DS_8** | 14904  14597  14538 | 30529  13567  0 * | 14986  24983  0 * | 14877  31401  0 * | 20330  21193  17697 | 25653  0  0 ** |
| **DS_9** | 10297  9384  9314 | 9657  16349  0 * | 10022  9603  8274 | 12508  20672  0 * | 12774  11640  11415 | 31383  14348  0 * |
| **DS_10** | 10266  9319  9640 | 23326  9881  0 * | 24725  0  0 ** | 14533  14517  12881 | 18596  19111  17441 | 30126  13468  0 * |

DS: Down syndrome patient. * aberrant ratio between peaks (~1:2) reflecting trisomy 21. ** Uninformative outcome (one allele present). Note that none of the additional markers annotated to chromosome 13 and 18 or other locus, showed aberrant profiles.

Figure A: Karyogram all Down syndrome (DS) patients

DS-2

DS-1


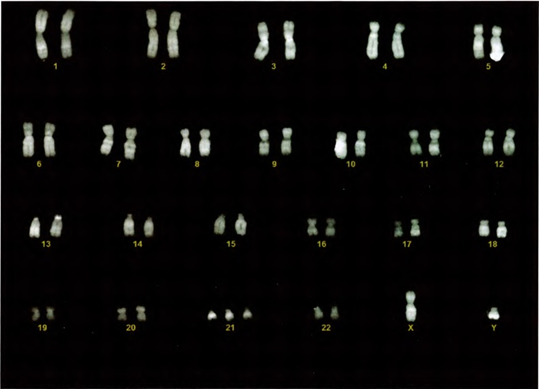


DS-4

DS-3


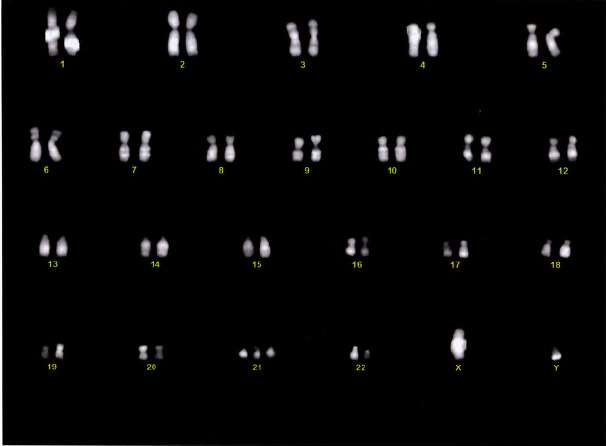


DS-6

DS-5


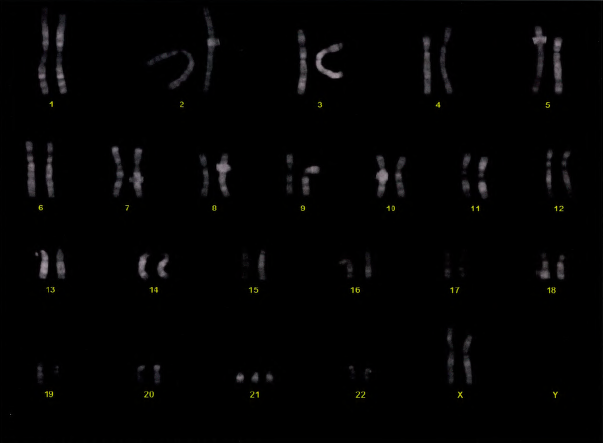

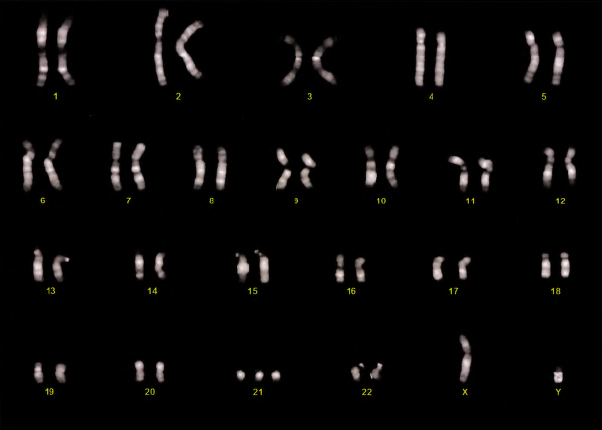


Figure A (continued): Karyogram all Down syndrome patients

DS-8

DS-7


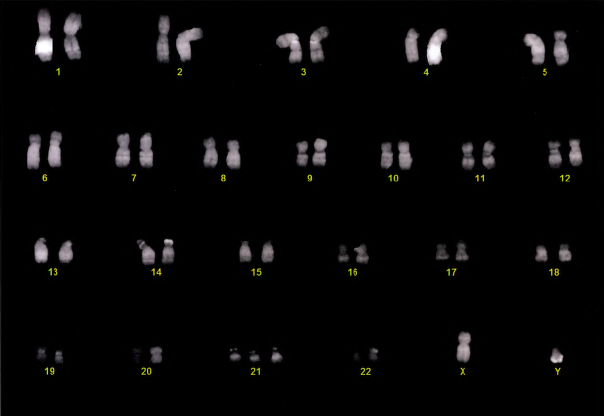

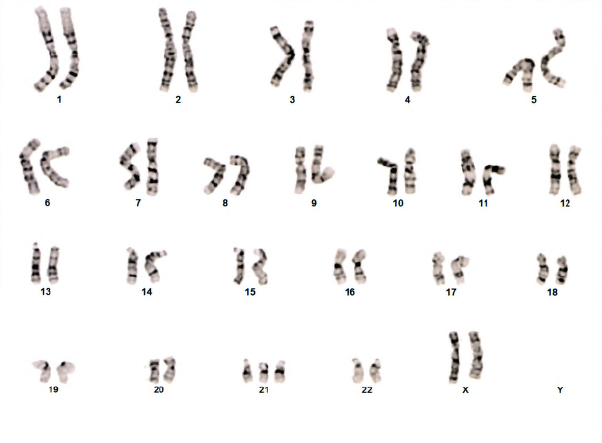


DS-10

DS-9


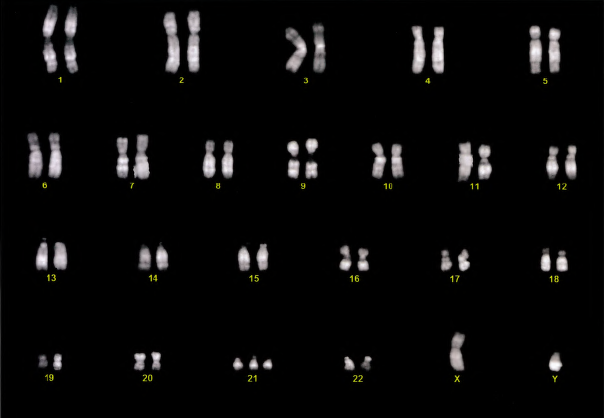

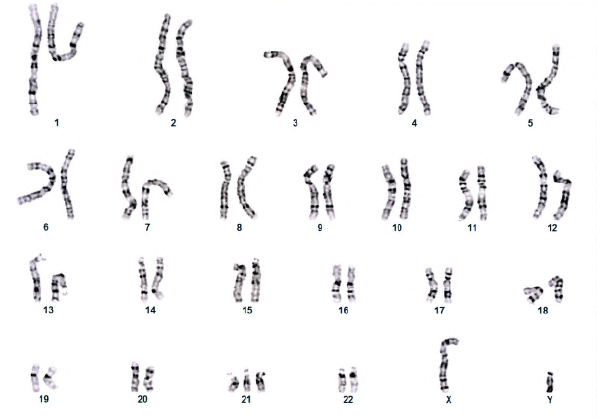


**R-code minfi script**

The following R script was used for detection of differentially methylated positions (DMPs) and regions (DMRs). Prior to the genome-wide association analyses the dataset was cleaned, i.e. probes annotated to the X and Y chromosome, SNPs , a-specific and probes prone for cross-hybridization were removed from the dataset[1, 2]. Further information on the script(s) available at the Bioconductor page, “minfi”(www.bioconductor.org/packages/release/bioc/html/minfi.html)

**#Read datafiles and remove -as you wish- XY, SNP, promiscuous probes**

RGset=read.450k.exp(,targets)

annotation=getAnnotation(RGset)

annotation.gr <- makeGRangesFromDataFrame(annotation, keep.extra.columns = T, start.field = "pos", end.field = "pos")

autosomes = annotation[!annotation$chr %in% c("chrX","chrY"), ]

GMset = preprocessQuantile(RGset, fixOutliers = FALSE, removeBadSamples = FALSE, quantileNormalize = TRUE, stratified = TRUE, mergeManifest = TRUE, sex = NULL, verbose = TRUE)

GMset.noXY = GMset[annotationreNames(GMset) %in% row.names(autosomes),] #Remove XY

**#Remove SNP with maf boundaries**

SNP_ANNO = getSnpInfo(GMset.noXY)

SNP_ANNO$CpG_maf[SNP_ANNO$CpG_maf < 0.05]=NA #Give boundaries

SNP_ANNO$Probe_maf[SNP_ANNO$Probe_maf < 0.05]=NA #Give boundaries

SNP_ANNO$SBE_maf[SNP_ANNO$SBE_maf < 0.05]=NA #Give boundaries

cpgsToKeep = intersect(which(is.na(SNP_ANNO$CpG_maf)),which(is.na(SNP_ANNO$SBE_maf)))

cpgsToKeep2 = intersect(which(is.na(SNP_ANNO$Probe_maf)),cpgsToKeep)

GMset.noXY.noSNPs = GMset.noXY[cpgsToKeep2,]

**#Remove population specific SNPS with set**

popprobes = read.csv("drivelocation\Polymorphic-CpGs-Illumina450k.csv", sep=",",header=T)

popprobes$EUR_AF[popprobes$EUR_AF < 0.05]=NA #Give boundaries

popprobes = popprobes[!is.na(popprobes$EUR_AF),]

GMset.noXY.noSNPs.noPOP = GMset.noXY.noSNPs[which(annotationreNames(GMset.noXY.noSNPs) %nin% popprobes$PROBE),]

**#Remove promiscuous probes**

promprobes = read.csv("drivelocation\Non-specific-probes-Illumina450k.csv", sep=",",header=T)

GMset.noXY.noSNPs.noPOP.noPP = GMset.noXY.noSNPs.noPOP[which(annotationreNames(GMset.noXY.noSNPs.noPOP) %nin% promprobes$TargetID),]

**#Betas (aka DMP)**

pd = pData(GMset.noXY.noSNPs.noPOP) #Select pData from desired GMset

beta = getBeta(GMset.noXY.noSNPs.noPOP) #Extract betas from desired GMset

beta.frame=as.data.frame(beta)

colnames(beta.frame) = pd$Sample_Group #Determine test and controls

beta.frame$Delta = rowMeans(beta.frame[,colnames(beta.frame)=="down"]) - rowMeans(beta.frame[,colnames(beta.frame)=="control"]) #Calculate Delta

annotation2=as.matrix(annotation)

tmp=data.frame(annotation2[match(row.names(beta.frame), row.names(annotation2)),]) #Get annotation data for probes

beta_delta=cbind(beta.frame,tmp) #Bind annotation to dmps

write.table(beta_delta,"drivelocation\Betas-xy-SNP.txt",sep="\t",col.names=T,row.names=T)

**#Celldistribution calculation**

targets$Sample_Name=as.character(targets$Sample_Name)

targets$Sex=as.character(targets$Sex)

targets$Basename=as.character(targets$Basename)

RGset_cell=read.450k.exp(,targets)

cell<-estimateCellCounts(RGset_cell, compositeCellType = "Blood", cellTypes = c("CD8T","CD4T", "NK","Bcell","Mono","Gran"), returnAll = T, meanPlot = TRUE, verbose = TRUE)

distribution=as.data.frame(cell$counts)

write.table(distribution,"drivelocation\Celldistribution.txt",sep="\t")

**#Celldistribution correction lmfit**

cd8t=as.numeric(distribution$CD8T) #Set variables

cd4t=as.numeric(distribution$CD4T)

nk=as.numeric(distribution$NK)

bcell=as.numeric(distribution$Bcell)

mono=as.numeric(distribution$Mono)

gran=as.numeric(distribution$Gran)

design.cor=model.matrix(~0+group+gender+cd8t+cd4t+nk+bcell+mono+gran,pd) #Make design matrix

cont.matrix.cor=makeContrasts(caseContr=(groupdown-groupcontrol), levels=design.cor) #Set contrasting groups

lmfit.cor <- lmFit(beta, design.cor, method="robust") #Lineair model correction, set method as desired (ignore the 50 or more warnings messages)

cor.lmfit=contrasts.fit(lmfit.cor,cont.matrix.cor)

cor.fit <- eBayes(cor.lmfit) #Bayes to moderate the standard deviations between genes

cor_fit.none=topTable(cor.fit, n=nrow(beta), adjust.method="none") #From here down is for output purposes as desired

cor_fit.BH=topTable(cor.fit, n=nrow(beta), adjust.method="BH")

cor_fit.BF=topTable(cor.fit, n=nrow(beta), adjust.method="bonferroni")

cor_fit=cbind(cor_fit.none, cor_fit.BH[,5], cor_fit.BF[,5])

colnames(cor_fit)[c(5,7,8)]=c("No Adjustment", "q-value", "Bonferroni")

tmp=data.frame(beta_delta[match(row.names(cor_fit), row.names(beta_delta)),])

cor_fit=cbind(cor_fit,tmp$Delta)

colnames(cor_fit)[colnames(cor_fit)=="tmp$Delta"] = "Delta"

tmp=data.frame(annotation2[match(row.names(cor_fit), row.names(annotation2)),])

cor.fit.pos=cbind(cor_fit,tmp)

write.table(cor.fit.pos,"drivelocation\Corrected_lmfit.txt", sep="\t",col.names=F,row.names=T) # write data to file

**#Bumps corrected (aka DMR)**

dmrs.bh.cor = bumphunter(object = GMset.noXY.noSNPs, design = model.matrix(~group+gender+cd8t+cd4t+nk+bcell+mono+gran), coef = 2, cutoff = 0.2, nullMethod = "bootstrap", type="Beta", B=500, smooth = T, smoothFunction = loessByCluster)

bumps.cor=dmrs.bh.cor$table

bumps.cor = subset(bumps.cor, bumps.cor$L > 2) #Bumps more than 2 probes

bumps.cor.gr <- makeGRangesFromDataFrame(bumps.cor, keep.extra.columns = TRUE)

bumps.cor.ng = annotation.gr[nearest(x = bumps.cor.gr, subject = annotation.gr),]

bumps2.cor = cbind(bumps.cor, Gene_start = as.data.frame(ranges(bumps.cor.ng))$start, Gene_end = as.data.frame(ranges(bumps.cor.ng))$end, Gene_Name = bumps.cor.ng$UCSC_RefGene_Name)

write.table(bumps2.cor,"drivelocation\Cor_Bumps.txt",sep="\t")

**References**

1. Aryee MJ, Jaffe AE, Corrada-Bravo H, Ladd-Acosta C, Feinberg AP, Hansen KD, et al. Minfi: a flexible and comprehensive Bioconductor package for the analysis of Infinium DNA methylation microarrays. Bioinformatics. 2014;30(10):1363-9. doi: 10.1093/bioinformatics/btu049. PubMed PMID: 24478339; PubMed Central PMCID: PMCPMC4016708.

2. Chen YA, Lemire M, Choufani S, Butcher DT, Grafodatskaya D, Zanke BW, et al. Discovery of cross-reactive probes and polymorphic CpGs in the Illumina Infinium HumanMethylation450 microarray. Epigenetics. 2013;8(2):203-9. doi: 10.4161/epi.23470. PubMed PMID: 23314698; PubMed Central PMCID: PMCPMC3592906.
