# Supplementary material for: Widespread domain-like perturbations of DNA methylation in whole blood of Down syndrome neonates
Source: PLoS One. 2018 Mar 30;13(3):e0194938. doi: 10.1371/journal.pone.0194938 (PMC5877863; doi:10.1371/journal.pone.0194938)
Supplement: S1 Fig — (a) Density plot raw and normalized (Quantile) data; (b) Plot principal component analysis (PCA), component 1 (20.2%) vs. component 2 (18.8%); component 3 (7.8%) and component 4 (6.3) are not shown. Colour annotation represents cases (red: Down syndrome) and controls (black healthy controls). X and Y chromosomes were excluded from analysis.; (c) Plot principal component analysis (PCA), component 1 (20.2%) vs. component 2 (18.8%); component 3 (7.8%) and component 4 (6.3) are not shown. Colour annotation represents genders (red: male, black: female); D and N represent Down syndrome and healthy controls, respectively. X and Y chromosomes were excluded from analysis. (DOC) [file pone.0194938.s004.doc]

**S1a Figure**

Beta

Beta

**S1b Figure**

**S1c Figure**
